# Where, When, and How? Integrating Spatiotemporal Cues in Cell Division

**DOI:** 10.1002/bies.70093

**Published:** 2025-11-27

**Authors:** Luca Cirillo, Hradini Konthalapalli, Claudio Alfieri, Jonathon Pines

**Affiliations:** ^1^ The Institute of Cancer Research London UK

## Abstract

To an external observer, the goal of cell division is evident from the very shape of the duplicated chromosomes. Cells, however, cannot see—they must proceed by groping in the dark, searching for their own DNA—and a series of sophisticated spatial mechanisms enables them to align and segregate their genetic material. Spatial organization is only part of the challenge: cell division is also a race against time—spending too little or too much time in mitosis can be equally detrimental to cell survival. Dividing cells must not only coordinate the movement of often dozens of chromosomes but must do so with precise timing. Yet, chromosome segregation occurs with remarkable accuracy. In this review, we highlight the role of mitotic chromosomes as a platform to integrate spatial and temporal cues to ensure their successful segregation.

## Graphical abstract

Accurate chromosome segregation during mitosis depends on the spatial and temporal regulation of spindle assembly, kinetochore‐microtubule attachment, and checkpoint signaling. Here, we highlight how mitotic chromosomes can serve as dynamic hubs that integrate molecular cues to coordinate spindle organization and timing, ensuring faithful genome segregation and maintaining genomic stability.

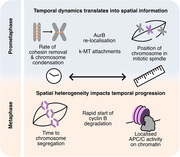

## 1 Introduction

**FIGURE 1:**
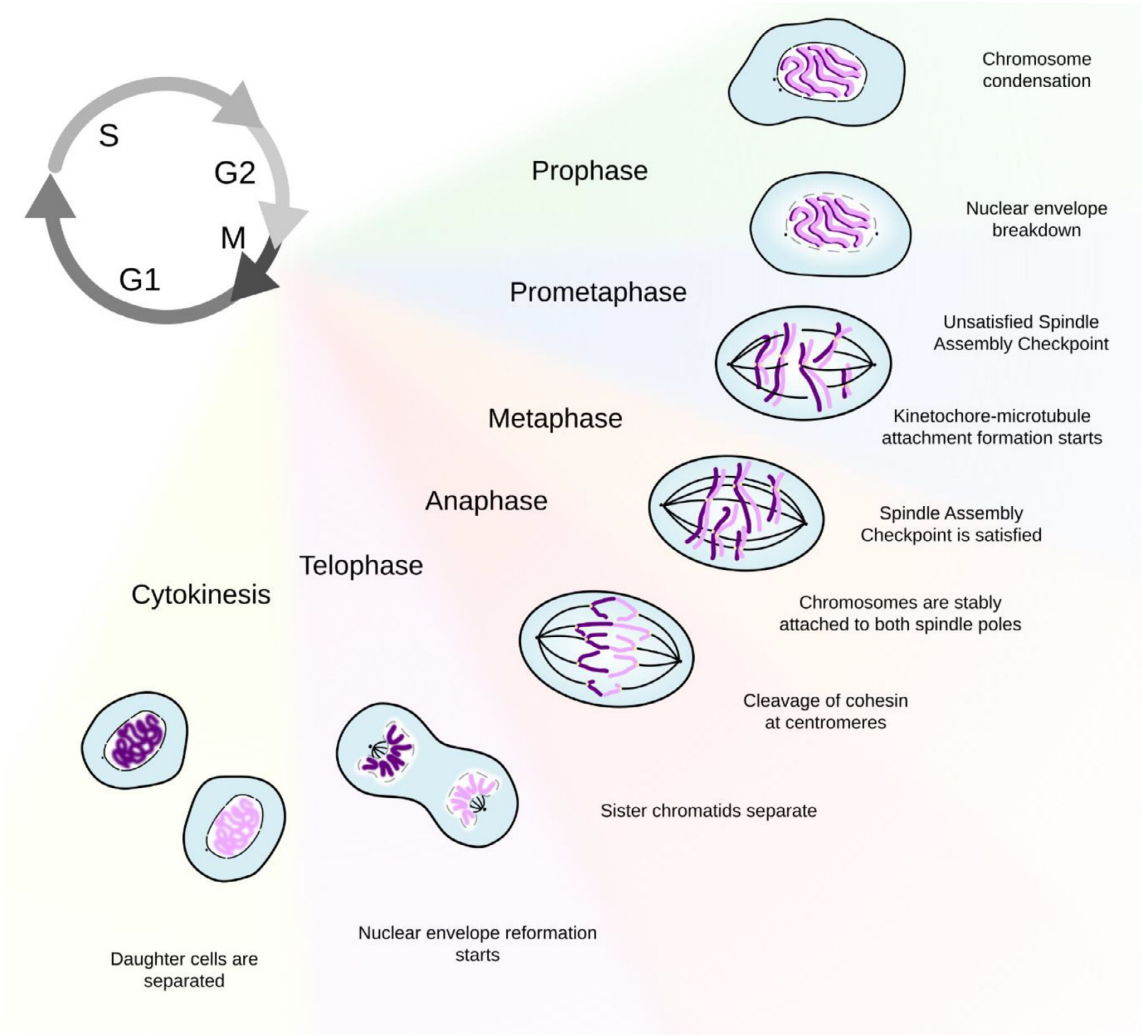
**Key events in mitosis**. To divide, a cell requires a major reorganization of its internal structures. This reorganization is so dramatic that cell division can be subdivided in six stages: prophase, prometaphase, metaphase, anaphase, telophase, and cytokinesis. The events between the start of chromosome condensation and nuclear envelope breakdown (NEBD) define prophase, when the duplicated centrosomes move to opposite sides of the cell and start forming the mitotic spindle. After NEBD, the prometaphase chromosomes are exposed to the cytoplasmic material and their kinetochores are captured by microtubules emanating from the mitotic spindle. Alignment of the chromosome to the spindle equator marks the end of prometaphase and the beginning of metaphase. Molecularly, this event coincides with the inactivation (satisfaction) of the SAC and the full activation of APC/C. Active APC/C targets for degradation the two inhibitors of separase, cyclin B1 and Securin. This leads to a drop in Cdk1 activity and to the separase‐dependent cleavage of cohesin at centromeres. Cohesin cleavage enables the segregation of chromatids to the opposite pole of the dividing cell, which defines anaphase. In the last steps of cell division, nuclei form around the chromosomes in the two daughter cells that are separated through cytokinesis.

Cell division is the sequence of events enabling a cell to segregate an identical copy of the genetic material to two daughter cells (Figure 1). Broadly speaking, the chromosome segregation machinery must comprise at least two components: a motor and a sensor. The motor is provided by the mitotic spindle, a cytoskeletal structure made of microtubules that dock to each sister chromatid at the level of the kinetochore (reviewed in [1]). The ability of microtubules to grow and shrink, alongside the actions of molecular motors and microtubule associated proteins (MAPs) allow the cell to exert pulling and pushing forces that the kinetochores convert into chromosome movements. Each chromatid has one kinetochore, which faces away from its sister such that each chromosome attaches to microtubules emanating from opposite poles of the mitotic spindle. The tension generated by microtubules pulling on a kinetochore stabilizes microtubule attachment. Thus, kinetochores actively contribute to the stabilization of microtubules and to the force‐generation machinery; they act as topological sensors to discriminate between correct and incorrect kinetochore‐microtubule attachments (reviewed in [1, 2, 3]).

Forces are only meaningful, however, in a coordinate system. In the dividing cell, the coordinate system involves both signals on the microtubule lattice, such as microtubule polarity and post‐translational modifications (PTMs), signals from the chromosomes and the kinetochores, such as the RanGTP gradient (reviewed in [4]) and Aurora B kinase localization (reviewed in [5]). The interplay between spindle forces and positional cues is sufficient to align individual chromosomes at the metaphase plate and ensure their equal segregation into daughter cells. Coordinating the movement of all chromosomes, however, requires centralized control. The cell cycle machinery fulfils this role, ensuring that each step of the cycle occurs in the correct order. Thus, in parallel to the spatial regulation of chromosome alignment and segregation, the dividing cell is also under strict temporal control. Central to the temporal regulation of mitosis is Cyclin‐Dependent Kinase 1 (Cdk1) that in complex with an activator cyclin (either cyclin A or B in higher eukaryotes) and a Cks (Cyclin‐dependent kinase regulatory subunit) protein, phosphorylates substrates crucial for cell division (reviewed in [6]). The mitotic exit program is initiated by the inactivation of Cdk1 [7, 8, 9], which is mediated by the ubiquitination and degradation of cyclin B by the Anaphase Promoting Complex/Cyclosome (APC/C) ubiquitin ligase [10, 11, 12]. APC/C is also central to chromosome segregation, because it unleashes separase, the proteolytic enzyme that cleaves the cohesin complex holding sister chromatids together [13, 14]. Left without a counterbalance, the forces exerted from opposite poles of the spindle are now free to pull sister chromatids apart, initiating the process of anaphase.

Both spatial and temporal control of mitosis are crucial for the equal segregation of genetic material, and the cell evolved multiple strategies to integrate spatial and temporal cues. The most studied of these is the Spindle Assembly Checkpoint (SAC), a surveillance mechanism activated by kinetochores that are unattached or incorrectly attached to the mitotic spindle (reviewed in [15, 16]). The SAC is inactivated (or satisfied) once all the kinetochores are stably attached to microtubules. Albeit less appreciated, cells have other opportunities to integrate spatial and temporal information. Here, we discuss how temporal processes such as the restructuring of mitotic chromatin may be used to regulate the position of the chromosomes during early mitosis. Later in mitosis the situation appears to reverse as localized activity of the APC/C is spatial information that the cells can use to pace mitotic events. From our discussion, chromosomes emerge as important signaling hubs that integrate spatial and temporal information in the dividing cell, a notion that challenges the original view of the chromosome as passive entities during mitosis.

We will focus primarily on somatic, symmetrical, cell division in higher eukaryotes, with examples from other organisms where relevant. We limit our discussion to the processes of chromosome alignment and segregation although other mechanisms such as spindle positioning, nuclear envelope breakdown (NEBD) and reformation, and cytokinesis may also represent signaling hubs to integrate spatial and temporal information.

## 2 How to Build a Mitotic Chromosome

During interphase, chromosomes are widely spread in the nucleus; single chromosomes are highly intertwined and indistinguishable without specialized techniques. The classical representation of a duplicated human chromosome is, however, that of a rod‐shaped chromatin fiber composed of two clearly defined sister chromatids with a single connecting region corresponding to the centromere. This configuration is adopted by chromosomes during cell division (refer to Paweletz, 2001 for an historical perspective [17]), and it is so highly condensed that its ultrastructure is almost inaccessible to direct visualization techniques and remains an area of active research [18, 19].

Two distinct pathways cooperate to transform a chromosome from its relaxed interphase state to a highly condensed state during mitosis: chromatid cohesion and chromosome condensation. Both pathways are under the control of remarkably similar protein complexes sharing a common evolutionary history: the cohesin and condensin complexes (reviewed in [20]). Cohesin activity in S‐phase is responsible for the linkage of duplicated chromatids, thus preceding the compaction role of condensin in mitotic prophase. At metaphase, condensin activity is responsible for the rod shape of chromatids, and the removal of cohesins from chromosome arms generates the well‐known X‐shape of human chromosomes.

In this section, we will briefly cover chromatid cohesion and chromosome condensation, and we will discuss the how these processes may represent signaling hubs to integrate temporal and spatial information in the mitotic cell.

### 2.1 Cohesin Removal

Through different modes of binding to chromatin, the cohesin complex plays essential roles in 3D genome organization, gene expression, and sister chromatid cohesion (reviewed in [21, 22]). For this latter function, the cohesin ring is proposed to entrap the two sister chromatids of a duplicated chromosome. Holding sister chromatids together is essential for their coordinated separation during mitosis; without this linkage, their distribution to opposite poles would occur randomly. Cohesin rings, however, must ultimately be removed to permit chromatid segregation.

**FIGURE 2:**
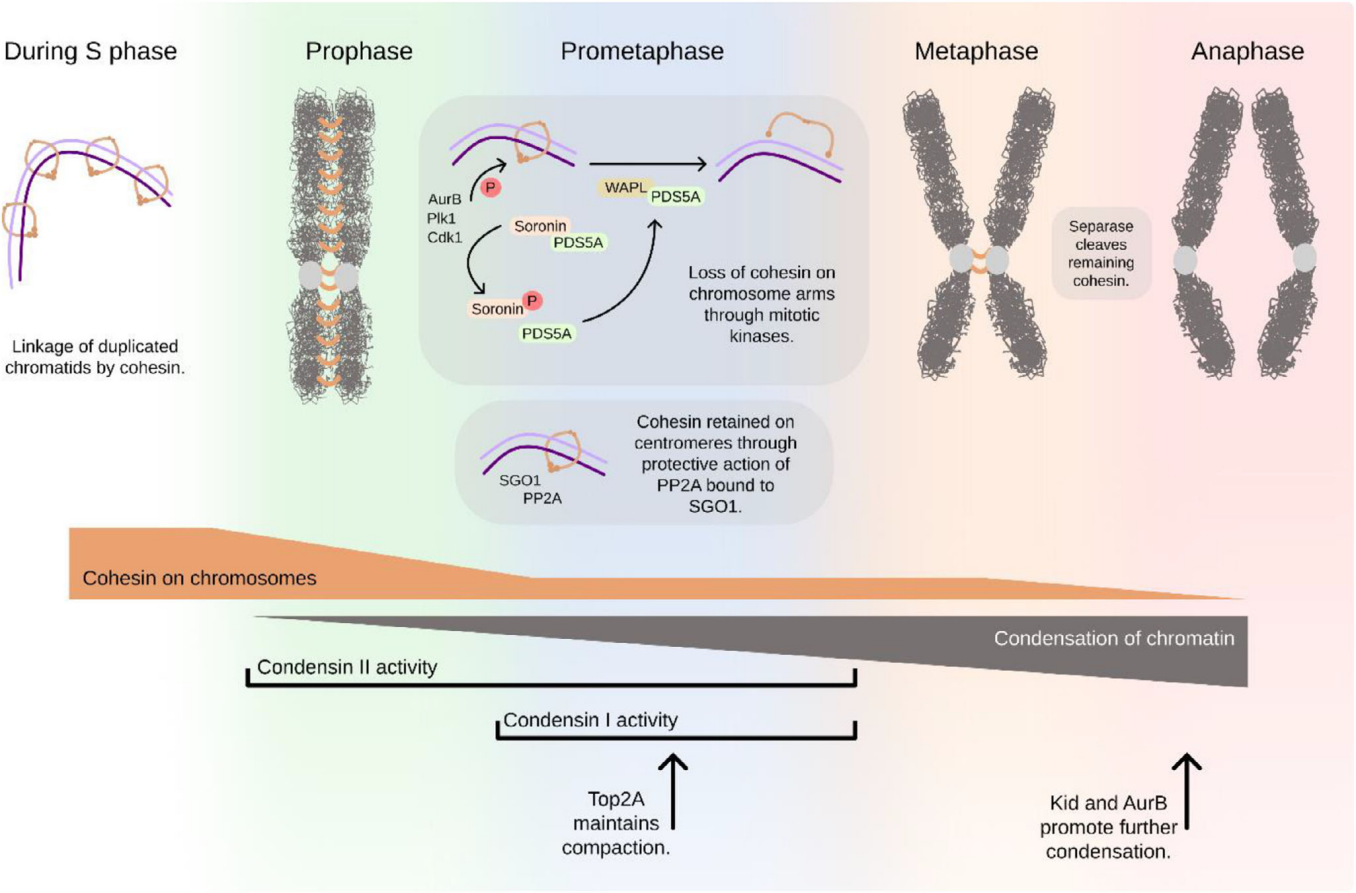
Chromatin reorganization may serve as a prometaphase stopwatch. Following DNA duplication in S phase, sister chromatids remain associated thanks to the action of cohesins. Cohesins must be removed during cell division to allow chromosome segregation. During prophase and prometaphase, the kinases Plk1, Cdk1, and Aurora B target several components of the cohesin complex. Phosphorylation of Sororin and SA2 are important to free up PDS5A, which can in turn interact with WAPL. The WAPL‐PDS5A complex triggers the opening of the cohesin ring and the release of entrapped DNA, resulting in a loss of cohesin at chromosome arms. At centromeres, PP2A and SGO1 protect cohesins from WAPL‐PDS5A activity. This residual population of cohesin requires the action of the protease separase which becomes active during metaphase owing to the degradation of its inhibitors securin and cyclin B.

In metazoans, cohesin removal occurs through two temporally distinct pathways. During prophase and prometaphase, the cohesin rings on chromosome arms are removed by the coordinated action of the mitotic kinases Plk1 (Polo like kinase 1), Aurora B, and Cdk1, which target Sororin and the cohesin component SA2 (Stromal antigen 2 [23, 24, 25, 26]). Phosphorylation of Sororin causes its dissociation from PDS5A (Precocious dissociation of sister's protein 5), which is then free to interact with WAPL (Wings apart‐like protein homolog). The WAPL–PDS5A complex triggers the opening of the cohesin ring and the release of entrapped DNA [27, 28, 29, 30]. A small fraction of cohesin remains associated with chromatin until metaphase, thanks to the protective action of Protein Phosphatase 2A (PP2A) bound to Shugoshin at centromeres (Figure 2) [26, 31, 32, 33]. This residual pool of cohesin at centromeres is sufficient to prevent premature chromosome segregation until metaphase, when separase cleaves one of the subunits of the cohesin ring (see below; [13, 14]). As a result of this dual pathway, cohesion at chromosome arms is lost during prophase and prometaphase, while centromeres remain connected until the end of metaphase.

### 2.2 Chromosome Condensation

In interphase, chromosomes are highly intertwined, forming a structure reminiscent of a ball of wool. Although this arrangement may appear random, the relative position of each chromosome within the nucleus is actively maintained (reviewed in [34, 35, 36]). Such nuclear organization is functionally important, as regulatory elements located hundreds of kilobases apart along the DNA can be positioned only nanometers away in three‐dimensional space. During mitosis, most of these long‐range interactions are lost, and chromosomes adopt a condensed configuration that favors short‐range inter‐chromosomal contacts [19]. Chromosome condensation occurs mainly thanks to the sequential action of condensin II (prophase and prometaphase) and condensin I (prometaphase) (Figure 2) [37, 38]. How condensin II becomes active at mitotic onset remains unclear, but it involves phosphorylation by Cdk1, Mps1 (Monopolar spindle kinase) and Plk1 [39, 40, 41], interaction with the phosphatase PP2A and MCPH1 (Microcephalin 1) [42, 43] and mitotic histone modification—at least in some non‐human models [44, 45]. Chromosome condensation is not mediated by condensins alone: topoisomerase II contributes to compaction during prometaphase [46, 47, 48], while Kid (a kinesin‐like DNA‐binding protein) and Aurora B promote further condensation in early anaphase [49, 50, 51].

Since the compression of chromosomes in prometaphase coincides with the prophase pathway of cohesin removal, it is likely that these processes are somewhat related. However, the exact interplay between condensin I, II, and cohesin, remains under debate [18, 19] (reviewed in [52]).

### 2.3 Integrating Chromosome Reorganization and Alignment

Chromosome reorganization is one of the most widely recognized hallmarks of cell division, to the point that the very term “mitosis” derives from the thread‐like shape of mitotic chromosomes (see [17, 53] for an historical perspective).

Neither cohesin removal from chromosome arms nor chromosome condensation have switch‐like dynamics but are instead gradual processes (Figure 2). Live‐cell microscopy shows that chromatin‐bound cohesin levels drop by approximately 70% from late G2 through to 6 min after nuclear envelope breakdown (NEBD), when they plateau at ∼10% of the original value until late metaphase [54, 55, 56]. As a result, cohesion between chromosome arms decreases progressively: during early prometaphase, chromatids remain paired, but by metaphase chromosomes have assumed their characteristic X‐shape. Similar to cohesin removal, chromosome condensation also proceeds gradually from early prophase until it peaks at anaphase onset, as revealed by both imaging techniques and Hi‐C (High‐throughput Chromosome Conformation Capture) [19, 57, 58]. Thus, both cohesin removal and chromosome condensation offer to the cell a natural “stopwatch” to measure time; but can the cell use this stopwatch (a temporal cue) to regulate the position of chromosomes (spatial information)? If this were the case, one would expect that artificially slowing down or speeding up the stopwatch would have functional consequences on cell organization and on subsequent cell cycle decisions.

When cohesin removal is delayed—most commonly through WAPL depletion—cells experience defects in chromosome alignment [59, 60]. These defects are linked primarily to the localization of Aurora B at centromeres [61, 62, 63, 64]. Mislocalized Aurora B results in defective kinetochore‐microtubule attachment, SAC satisfaction, and mitotic arrest. These data support the notion that the gradual loss of chromatid cohesion contributes to proper kinetochore–microtubule attachment; thereby coupling a temporal cue (the rate of cohesin removal) to a spatial outcome (accurate chromosome alignment) (Figure 2).

Defects in chromosome condensation can also result in chromosome misalignment and SAC satisfaction. In Drosophila mutants of the condensin I subunit Cap‐G (Chromosome associated protein G), chromosome condensation is compromised in early mitosis and chromosome alignment is impaired [65]. In human cells, late condensing chromosomes remain unaligned and activate the SAC [66]. These chromosomes fail to localize Aurora B at centromeres making it likely that defective kinetochore‐microtubule attachment is responsible for chromosome misalignment, a conclusion supported by a study on Acute Lymphoblastic Leukemia cells that found chromosome hypocondensation and mislocalized Aurora B [67]. Thus, the rate of chromosome condensation may also represent temporal information that the cells use to regulate the spatial organization of chromosomes. The link between delayed chromosome condensation and SAC satisfaction is not universally accepted; however, as one study reported that defective chromosome condensation does not activate the SAC [68], resulting instead in severe chromosome segregation defects.

That reduced Aurora B kinase activity at kinetochores triggers the SAC may seem counterintuitive given that global inhibition of Aurora B abolishes the SAC [69, 70]. But neither defective cohesin removal nor chromosome condensation leads to full inhibition of the kinase, instead it is mislocalized. When cohesin removal is perturbed, Aurora B remains at the kinetochore instead of relocating at centromeres, which induces sustained SAC signaling [61, 62, 63, 64]. A similar mechanism has been reported for defective chromosome condensation [66, 67].

In parallel to loss of cohesin, chromatin is compacted through the activity of Condensin II and Condensin I, a process that starts in prophase and culminates in early anaphase. Topoisomerase IIa (Top2A) contributes both to chromosome condensation and sister chromatid cohesion thanks to its decatenating activity. Both cohesin removal and chromatin condensation are gradual processes, offering cells a natural “stopwatch” that can be used to integrate temporal information with the spatial organization of chromosomes.

Since both cohesin removal and chromosome condensation converge on Aurora B regulation, and because cohesin removal is a prerequisite for correct chromosome condensation [19], one may question how far these two mechanisms represent different pathways rather than different aspects of the same one. However, WAPL depletion in human cells delays cohesin removal and results in chromosome segregation defects, but it does not alter the localization of condensin [60] indicating that cohesin removal contributes to Aurora B centromeric localization independently from condensin.

In summary, the restructuring of chromosomes during mitosis may represent a signaling hub where the cells use temporal information (the rate of cohesin removal and chromosome condensation) to achieve the correct spatial organization of chromosomes. Even though cohesin removal and chromosome condensation are deeply intertwined processes, they may contribute independently to chromosome alignment by establishing stable kinetochore‐microtubule attachments.

## 3 APC/C Activity

Progress through mitosis is directly or indirectly controlled by the kinase Cdk1. A sudden rise in Cdk1 activity drives mitotic entry [71, 72] (reviewed in [73]), while its inactivation following SAC satisfaction leads to mitotic exit [7, 74, 75] (reviewed in [76]). Central to mitotic exit is the APC/C ubiquitin ligase (reviewed in [77, 78]) that promotes the degradation of the Cdk1 activators cyclin A and B.

**FIGURE 3:**
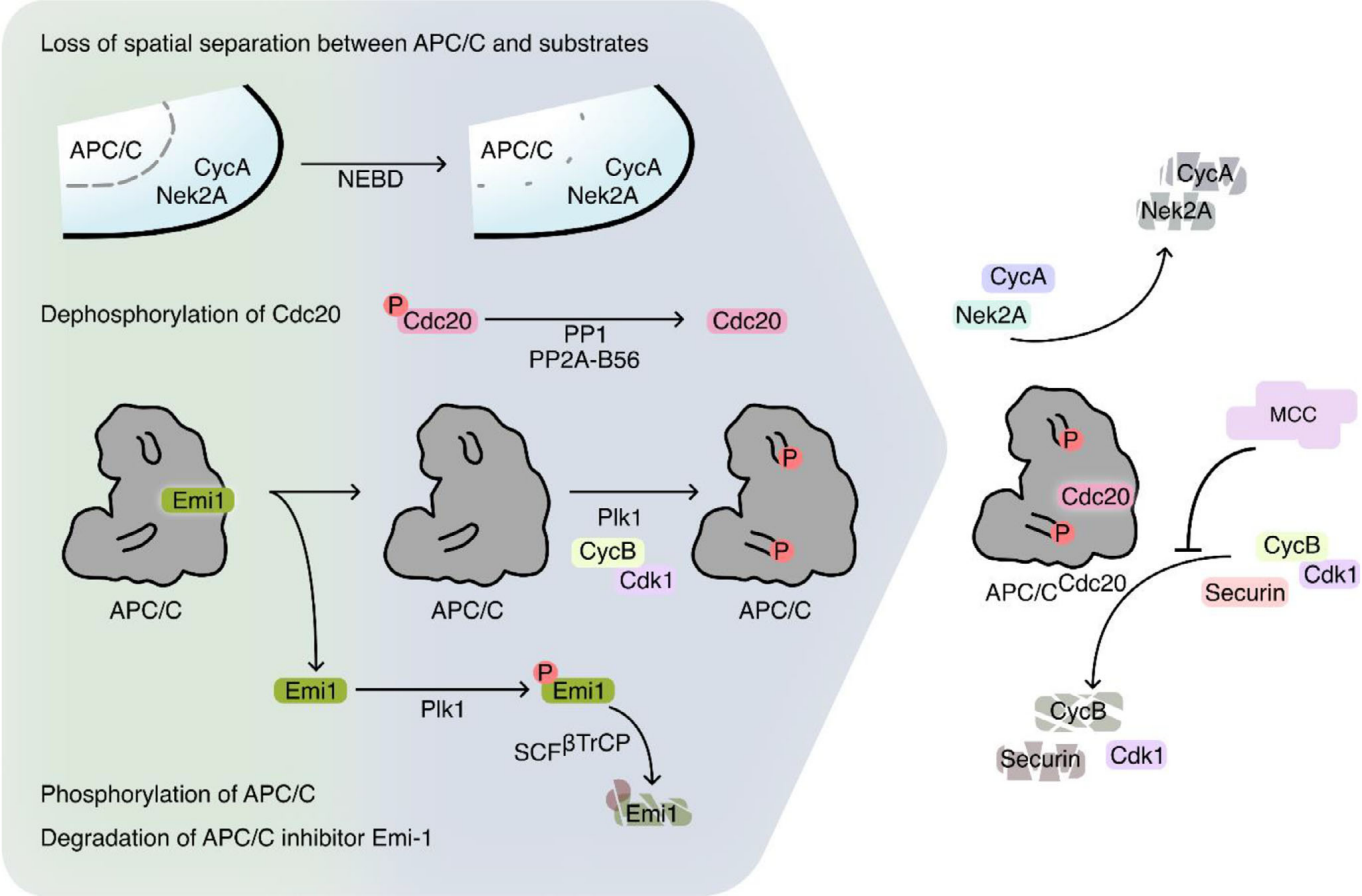
**APC/C activation and substrate selectivity after NEBD**. Four mechanisms lead to the activation of APC/C during cell division. First, NEBD leads to loss of spatial separation between APC/C and certain substrates, such as a subpopulation of Cyclin A and Nek2A. Second, Cdk1 and Plk1 promote both the degradation of the APC/C inhibitor Emi1 and the structural reorganization of the APC/C to accommodate binding of its coactivator Cdc20. APC/C does not reach full activity until metaphase due to the presence of the MCC that inhibits the degradation of certain APC/C substrates.

APC/C activation coincides with NEBD and depends on four converging mechanisms (Figure 3): loss of spatial separation between APC/C and its substrates [79], degradation of the APC/C inhibitor Emi‐1 (Early Mitotic Inhibitor 1 [80, 81]), phosphorylation of APC/C itself [82, 83], and the de‐phosphorylation of the APC/C co‐activator Cdc20 (Cell Division Cycle 20) [83, 84]. The activating phosphorylations on APC/C are regulated by Cdk1 [82, 85] and Plk1 [86, 87]. This creates a negative feedback loop in which Cdk1 promotes its own inactivation by stimulating the ubiquitylation and degradation of its cyclin partners [82, 88] (reviewed in [77, 78])

During mitosis, the main inhibitor of APC/C is the mitotic checkpoint complex (MCC), a hetero‐tetramer generated by the SAC (reviewed in [15, 16]). Not all APC/C substrates are equally sensitive to MCC inhibition. Some can bypass MCC‐mediated suppression and are degraded even in the presence of an active SAC, whereas others remain strongly protected until checkpoint silencing [89, 90, 91, 92]. This substrate selectivity is essential for ordering mitotic events, including chromosome alignment and segregation. Thus, the catalytic activity of the APC/C could represent a temporal cue, that functions as a timer for mitotic progression. This possibility has previously been explored in the context of cyclin A degradation during prometaphase. The progressive ubiquitylation and degradation of cyclin A during prometaphase triggers a gradual stabilization of kinetochore‐microtubule attachment, thus contributing to chromosome alignment [93, 94] (reviewed in [95]).

Moreover, APC/C activity may not act solely as a temporal regulator. Emerging evidence suggests that APC/C is also subject to spatial regulation, providing positional cues that influence mitotic timing [74, 96, 97].

In this section, we will explore the APC/C as a signaling hub integrating both spatial and temporal information during cell division. We will focus on the spatial regulation of APC/C and its potential role as a positional cue in mitotic progression. The role of APC/C in regulating chromosome alignment via cyclin A degradation has been reviewed elsewhere [95] and falls beyond the scope of this review.

### 3.1 Localized Activity of APC/C

Traditionally, APC/C activity has been viewed as homogeneous throughout the cell, since its subcellular localization appears diffuse, with only weak enrichment at centrosomes and the spindle [87, 98, 99]. However, several observations contradict this idea. Live‐cell imaging of fluorescently‐tagged human cyclin B1 reveals that its degradation begins on the spindle and centrosomes before spreading into the cytoplasm [7, 74]. Similarly, in Drosophila embryos cyclin B1 disappears first from the centrosomes and spindle and only later from the surrounding cytoplasm [96]. Biochemical fractionation of HeLa cells further supports this spatial heterogeneity because chromosome‐bound APC/C appears more active than its cytoplasmic counterpart [97].

One intriguing possibility is that the spatial regulation of the APC/C may represent only one aspect of general compartmentalization of the dividing cell. In our experiments we observed APC/C‐cyclin B1 complexes in the spindle apparatus (centrosomes, spindle, and chromosomes) but much less in the surrounding cytoplasm, and we put forward a model where the nucleosomes induce the initial binding between APC/C and cyclin B1, which can then travel together to other locations [74]. Fewer APC/C‐cyclin B1 complexes in the cytoplasm can be explained by a cytosolic mechanism that actively separates cyclin B1 from APC/C, or by some sort of barrier impeding APC/C‐cyclin B1 to exit the spindle area. In the first hypothesis, two pathways could remove cyclin B1 from the APC/C or prevent its binding in the cytoplasm: an especially active cytoplasmic form of the proteasome, or a residual pool of the MCC. It is difficult to reconcile a role of the proteasome in separating cyclin B1 from the APC/C with the delayed degradation of cyclin B1 in the cytoplasm. Moreover, we observed no difference in the amount of APC/C‐cyclin B1 complexes in the cytoplasm even under proteasome inhibition [74]. We also excluded a contribution of a residual of MCC pool blocking cyclin B1 binding to the APC/C because a mutant of cyclin B1 that cannot bind to chromosomes is still delayed in its degradation even when the SAC is abolished. Thus, neither localized proteasomal activity nor residual MCC complexes can explain the reduced number of APC/C‐cyclin B1 complexes in the cytoplasm in human cells.

The alternative hypothesis is that APC/C‐cyclin B1 complexes have difficulty diffusing into the cytoplasm. Historical experiments fusing HeLa cells showed that nuclei in a common cytoplasm tend to synchronize their cell cycle, leading to the idea that cell cycle control may be mediated by freely diffusible regulators [100]. However, asynchronous cell divisions in a shared cytoplasm occur in mammalian cells exposed to DNA damage or with unattached kinetochores [101, 102, 103]. In physiological conditions, the nuclei of the cricket *Gryllus bimaculatus* embryo undergo asynchronous cell division despite the syncytial nature of the embryo [104], and similar phenotypes exists in some fungi [105, 106] and apicomplexa [107]. Moreover, in a cell with two spindles, an unattached kinetochore in one spindle is unable to prevent anaphase in an adjacent spindle more than 20 µm away, likely due to the dilution of the MCC [101, 103]. These data could support the notion that at least some cell cycle regulators cannot diffuse freely outside the spindle apparatus. What could restrict the diffusion of molecules between the spindle apparatus and the surrounding cytoplasm remains unclear. Mechanisms that have been suggested to limit diffusion between the spindle apparatus and the cytoplasm include the persistence of nuclear envelope membranes around the spindle, and/or distinct biophysical properties of the mitotic spindle [108, 109, 110]. However, the reason for cell cycle asynchrony appears to vary widely according to the organism under investigation, arguing against an evolutionary conserved entity that separates the spindle from the cytoplasm. For example, in the filamentous fungus *Ashbya gossypii* the RNA binding protein Whi3 targets cyclin transcripts to some nuclei but not others [111, 112, 113], while in the apicomplex *Plasmodium falciparum* limiting resources of the host cell are responsible for asynchronous cell cycles [107].

Overall, these findings support the idea that APC/C activity is not uniform throughout mitotic cells. One intriguing possibility is that the compartmentalization of APC/C activity is only an aspect of a yet to be discovered organization of the dividing cell.

### 3.2 Why is APC/C Activity Localized?

A fascinating question is the physiological function of APC/C localization. Xenopus extracts continue cell cycle oscillations even in the absence of DNA [114, 115, 116, 117], indicating that localized APC/C activity at chromosomes is dispensable. Yet, hints of spatially regulated APC/C activity span across different model systems [96, 97], and decoupling cyclin B1 from chromosome‐bound APC/C delays its degradation and leads to genomic instability [74]. Thus, albeit not strictly required for cell cycle progression, localized activity of the APC/C contributes to the fidelity of chromosome segregation. One mechanistic explanation is that chromosomes catalyze the removal of separase inhibitors where separase activity is needed the most: cells would need to rapidly remove cyclin B1 and securin from chromosomes to relieve separase inhibition and allow rapid cleavage of centromeric cohesins [118, 119, 120].

Cell size is another, non‐mutually exclusive explanation for why APC/C activity is tied to chromosomes. Spatial constraints on APC/C activity may become increasingly relevant as the cell increases in size. Large cells, such as oocytes, fertilized eggs and those in the early embryo, must address two issues: they need to synchronize meiotic or mitotic events over relatively large distances, and they risk diluting active molecules in a large cytoplasmic volume. Focusing APC/C activity to a particular subcellular structure may contribute to the solution of both these problems.

Synchronizing anaphase onset across the entire cell is particularly demanding. In Xenopus extracts and Drosophila embryos, Cdk1 activity propagates as a phase wave, where locally activated Cdk1 molecules stimulate their neighbors in a positive feedback loop, creating a wavefront that moves faster than simple diffusion [121] (reviewed in [122]). Under metastable conditions, the phase wave becomes a trigger wave, which spreads at a constant speed and is relatively resistant to perturbations [123, 124]. Interestingly, Cdk1 inactivation also appears to follow a wave‐like pattern, though this process is much less understood than its activation. One hypothesis is that chromosomes provide a platform to initiate a wave of Cdk1 inactivation at mitotic exit. In this view, nucleosome‐mediated activation of APC/C could act as an “anaphase‐go” signal emanating from mitotic chromatin and propagating throughout the cell to synchronize the onset of anaphase. The existence of such a signal was proposed in 1997, based on cell fusion experiments that showed if two spindles share the same cytoplasm, when one spindle enters anaphase the second spindle follows, regardless of whether it has aligned its chromosomes [103].

Spatial regulation of APC/C might also serve a more physical purpose to prevent dilution of the complex in the cytoplasm—which is particularly relevant in large cells where the volume ratio between chromosomes and cytoplasm strongly favors the latter. Evidence from early *C. elegans* embryos shows that the strength of the SAC inversely correlates with cell size [125, 126], demonstrating how dilution of mitotic regulators becomes a limiting factor in large cells. Unlike the SAC [127, 128, 129, 130], APC/C activity is essential for mitotic progression in animals, thus cells may have evolved mechanisms to concentrate APC/C near chromosomes, ensuring locally high activity even when the cytoplasmic volume is large.

In summary, spatial regulation of the APC/C contributes to genome stability and it is likely to be important in large cells, where coordinating mitotic exit and maintaining sufficient APC/C activity pose unique challenges. Dissecting the contribution of local APC/C activity has been difficult, as perturbing such a large and essential complex at endogenous levels often compromises cell viability. We are only beginning to understand how APC/C is spatially regulated during mitosis, and future work will be needed to determine how this regulation contributes to the fidelity of cell division.

### 3.3 Chromosome Segregation

In our recent work, we began to dissect the molecular nature of APC/C spatial localization. We found that both APC/C and its substrate cyclin B1 directly bind to nucleosomes, and that this interaction is crucial for the timely degradation of cyclin B1 [74]. As such, APC/C may act as a hub that integrates spatial information (its localization at chromosomes) with temporal information inherently linked to cyclin B1 degradation, which in turn determines the length of metaphase and the timing of chromosome segregation.

Although it is tempting to view APC/C as a node of spatio‐temporal integration, directly testing this idea will require altering APC/C localization in living cells. One potential approach is to deplete subunits of the SKA (spindle‐ and kinetochore‐associated) complex, which contribute to APC/C loading onto chromatin [97, 131] (reviewed in [132]). However, this strategy is complicated by the pleiotropic roles of the SKA complex [133]. It is also possible that other genes contribute to APC/C chromatin binding; for instance, WDR5 (WD repeat‐containing protein 5) has been reported to localize APC/C to chromatin in postmitotic cells [134]. Alternatively, one could inhibit the stimulatory effect of nucleosomes on the APC/C, although the molecular mechanism by which chromosomes promote APC/C activity remains unclear.

Chromatin binding may not be the only determinant of APC/C spatial regulation. Local inhibition has been proposed at centrosomes, mediated by reported residual levels of the APC/C inhibitor Emi1 and the phosphatase PP2A [135, 136, 137, 138]. Moreover, PLEKHA5 (Pleckstrin homology domain‐containing family A member 5) is reported to enhance APC/C activity at microtubules, though the molecular mechanism remains unclear [139]. Future research should focus on elucidating the mechanisms by which APC/C activity is modulated to determine whether APC/C integrates spatial and temporal cues.

## 4 Conclusion and Perspective

Chromosomes have famously been described as passive entities during cell division [140], not contributing to the tide of pushing and pulling forces generated by the mitotic spindle. This view originated from the general transcriptional silencing that occurs during mitosis that largely prevents chromosomes from expressing their genetic content (reviewed in [141]). Here, we propose a chromosome‐centric view of cell division in which chromosomes are anything but passive spectators of mitosis. We speculate that chromosomes may serve as signaling hubs that integrate spatial and temporal information within dividing cells. It is worth remembering here that chromosomes direct spindle assembly through the RanGTP gradient and by localizing the activity of Aurora B (reviewed in [4, 142]).

**FIGURE 4:**
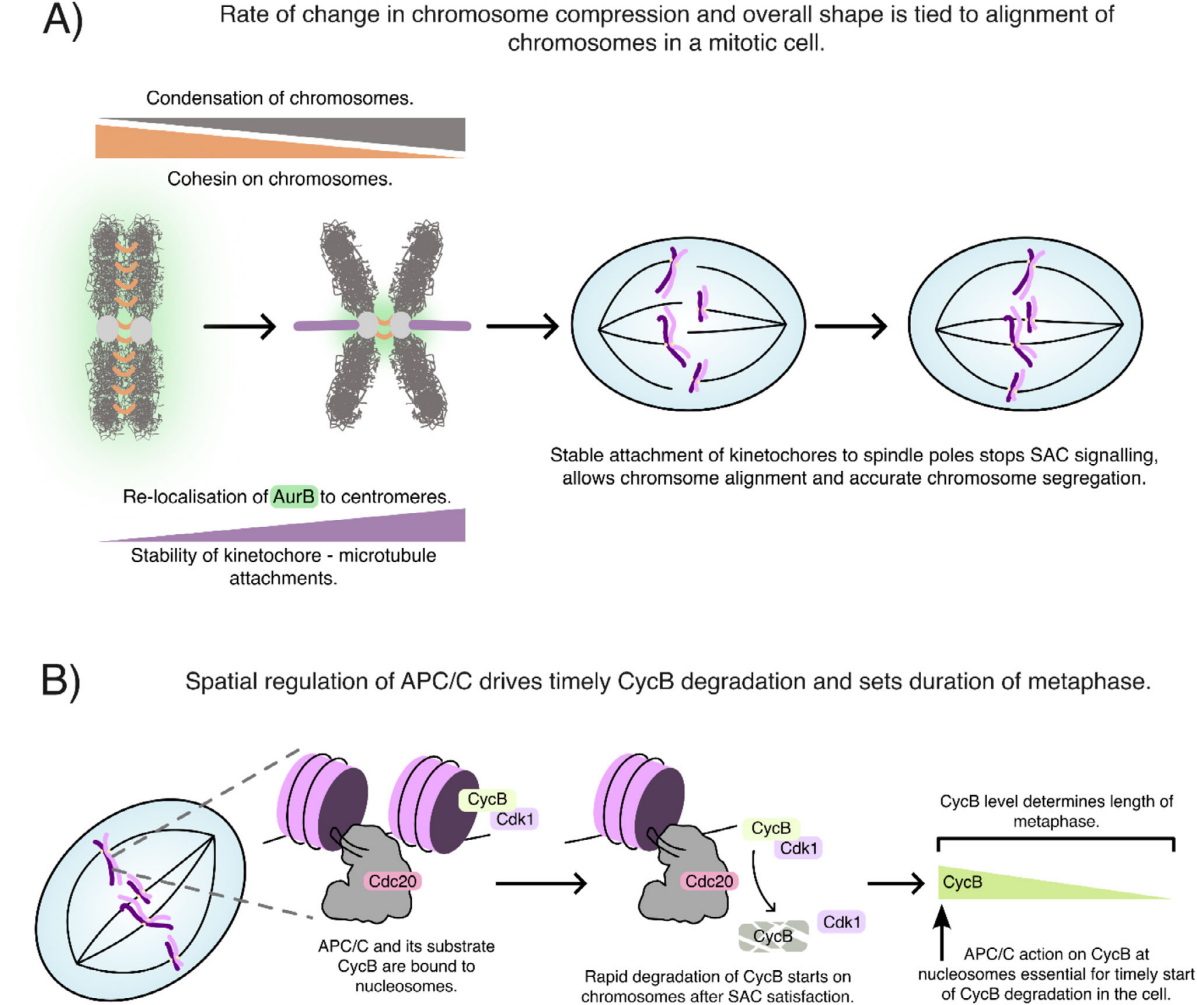
**Chromosomes as hubs to integrate spatiotemporal cues in mitosis**. (A) Chromosomes translate temporal information from their levels of condensation and loss of chromatid cohesion into a spatial cue for chromosome alignment. In both cases this may happen thanks to the centromeric relocalization of Aurora B, which promote stable kinetochore‐microtubule attachment. (B) High activity of APC/C on chromatin may regulate the timing of mitotic exit by promoting efficient removal of cyclin B1.

During prometaphase temporally defined processes such as cohesin removal from chromosome arms and chromosome condensation may translate into spatial information on a chromosome's position within the mitotic spindle. We propose that the gradual removal of cohesin and chromosome condensation progressively relocate Aurora B to centromeres, allowing the formation of stable kinetochore‐microtubule attachments. Early in mitosis, incomplete cohesin removal or insufficient condensation may favor kinetochore fiber turnover, promoting error correction. Later, as these processes advance, they may lead to stable kinetochore–microtubule attachments, which in turn promote SAC inactivation and metaphase onset (Figure 4). Whether cells use chromosome condensation or cohesin removal as intrinsic timers to regulate chromosome alignment remains to be tested. It would be particularly informative to measure kinetochore–microtubule stability under conditions where chromosome condensation or cohesin removal is impaired, and to test whether artificially stabilizing kinetochore–microtubule attachments can rescue delayed chromosome restructuring. Similar experiments have been performed in support of a model in which cyclin A degradation progressively increases kinetochore–microtubule stability during mitosis [93, 94]. Since recent studies have found a link between cyclin A degradation and cohesin removal from chromosome arms, it is possible that the effects on kinetochore fibers attributed to cohesin removal represent another aspect of cyclin A degradation [28].

If, during prometaphase, time instructs space, the situation appears reversed following SAC inactivation, when spatial control may directly impact temporal progression. The spatial regulation of the APC/C is central to this interpretation, as accumulating evidence supports the idea of localized APC/C activity that is crucial for promoting chromosome segregation in anaphase (Figure 4) [74, 97, 134, 139]. Testing this model remains challenging due to the difficulty of visualizing active APC/C, expressing APC/C mutants, and directly measuring its activity. Defining what constitutes active APC/C is itself challenging. For example, the traditional view that APC/C must be hyperphosphorylated to be active may be incomplete: the Gorbsky laboratory found that hypophosphorylated APC/C in chromatin fractions appears more active than the hyperphosphorylated form in the cytoplasm [97]. It is possible that the hypothetical “anaphase‐go” signal is a specific form of post‐translationally modified APC/C (see above). In addition to the difficulty in defining active APC/C, measuring APC/C activity is also not trivial, since this may differ toward different substrates. Notably, APC/C degrades phosphorylated securin in the cytoplasm earlier than unphosphorylated securin at the chromatin, highlighting how spatial regulation may change according to the substrate under examination [143].

In this review, we limited our discussion to chromosome alignment and segregation. However, cells may employ other mechanisms as internal clocks or spatial rulers to regulate key cell cycle events. One example could be the role of Aurora B in nuclear envelope reformation, but it remains unclear whether nuclear reformation following mitotic exit is governed by the spatial distribution of an Aurora B gradient and/or by the temporal cues of the cell cycle machinery (reviewed in [142]). Other, less‐explored spatial aspects of cell division may also contribute to temporal regulation. Among these, the local concentration of ions (such as Ca^2^⁺) and the possible presence of a diffusion barrier between the spindle apparatus and the surrounding cytoplasm could be particularly important (reviewed in [144]). Notably, positively charged ions contribute to chromosome condensation and may thus serve as spatial signals that modulate the timing of chromosome condensation [145, 146, 147].

In conclusion, chromosomes are not just passive spectator, but the very fulcrum of the spatiotemporal regulation of cell division. Why evolution selected chromosomes as a platform to integrate different signals is easily explained by the need to concentrate mitotic regulators (and their control) which would otherwise dilute in a vast cytoplasmic ocean. These mechanisms may be particularly important in large cells, and may help explain puzzling phenomena like asynchronous nuclear division in syncytial systems [104, 105, 111].

## Author Contributions

**L.C**.: Writing, original draft preparation, and editing. **H.K**.: figure design and editing. **C.A**. editing. **J.P**. writing and editing.

## Conflicts of Interest

The authors declare no competing interests.

## Data Availability

Data sharing is not applicable to this article as no datasets were generated or analyzed.
